# Combining next-generation pyrosequencing with microarray for large scale expression analysis in non-model species

**DOI:** 10.1186/1471-2164-10-555

**Published:** 2009-11-24

**Authors:** Diana Bellin, Alberto Ferrarini, Antonio Chimento, Olaf Kaiser, Natasha Levenkova, Pascal Bouffard, Massimo Delledonne

**Affiliations:** 1 Department of Biotechnology, University of Verona, Strada le Grazie 15, 37134 Verona, Italy; 2 Roche Diagnostics GmbH, Nonnenwald 2, 82377 Penzberg, Germany; 3 454 Life Sciences, 1 Commercial Street, Branford, CT 06405, USA

## Abstract

**Background:**

The next generation sequencing technologies provide new options to characterize the transcriptome and to develop affordable tools for functional genomics. We describe here an innovative approach for this purpose and demonstrate its potential also for non-model species.

**Results:**

The method we developed is based on 454 sequencing of 3' cDNA fragments from a normalized library constructed from pooled RNAs to generate, through *de novo *reads assembly, a large catalog of unique transcripts in organisms for which a comprehensive collection of transcripts or the complete genome sequence, is not available. This "virtual transcriptome" provides extensive coverage depth, and can be used for the setting up of a comprehensive microarray based expression analysis. We evaluated the potential of this approach by monitoring gene expression during berry maturation in *Vitis vinifera *as if no other sequence information was available for this species. The microarray designed on the berries' transcriptome derived from half of a 454 run detected the expression of 19,609 genes, and proved to be more informative than one of the most comprehensive grape microarrays available to date, the GrapeArray 1.2 developed by the Italian-French Public Consortium for Grapevine Genome Characterization, which could detect the expression of 15,556 genes in the same samples.

**Conclusion:**

This approach provides a powerful method to rapidly build up an extensive catalog of unique transcripts that can be successfully used to develop a microarray for large scale analysis of gene expression in any species, without the need for prior sequence knowledge.

## Background

Global analysis of gene expression is one of the most used tools in functional genomics. Hybridization to DNA microarrays is currently a standard method, but its application is limited to organisms for which the complete genome sequence or a large cDNA collection is available.

For biological systems that lack the sequence information necessary for development of microarrays, several alternative technologies based on cDNA fragment analysis or cDNA sequencing have been developed. The most successful and widespread of these is certainly cDNA-AFLP transcription profiling, which has so far represented one of the most robust and sensitive technologies for gene discovery on the basis of fragment detection [1,2]. However, this technique has several drawbacks. It involves a time-consuming and labor intensive series of PCR reactions and purification of resulting differentially expressed bands from gels followed by amplification and subsequent cloning and sequencing, it has a high rate of false positive (co-migrating) bands and, finally, it covers no more than 60-65% of the transcriptome due to the lack of restriction enzyme sites on the remaining cDNAs.

Tag based methods were developed to overcome these limitations, including serial analysis of gene expression (SAGE) [3] and massively parallel signature sequencing (MPSS) [4]. These high-throughput approaches can provide precise digital gene expression levels, but transcript abundance is derived from counting tags mapping to already known loci, thus requiring a reference genome.

Ultra-high throughput sequencing of the transcriptome is emerging as a powerful and attractive alternative technology for expression profiling. The RNA-Seq (RNA sequencing) approach to identify and quantify transcripts has already been applied with success to *Saccharomyces cerevisiae*, *Schizosaccaromyces pombe*, *Arabidopsis thaliana*, mouse and human cells [5-11] making use of the different deep sequencing technologies available to date: Illumina Genome Analyzer, Applied Biosystems SOLiD and Roche 454 Life Science. Also with this approach, the resulting sequence reads need to be individually mapped to a reference genome and counted to obtain the number and density of reads corresponding to RNA from each known exon. These data are also of great value to improve, validate and refine gene models on genomic sequences, and can identify new candidate genes. In *Vitis vinifera*, for example, integration of RNA-Seq data on the corresponding genomic sequence led to the identification of several genes that had been missed by the automatic annotation procedure in a genome that is already very well annotated [12]. Furthermore, always in model species, the RNA-Seq approach has the potential to overcome microarray limitations related to cross hybridization and difficult quantification of low abundance species, and provides gene expression information with a greater dynamic range [13]. Nevertheless, this technology still presents limitations, as the short reads require genomic or extensive cDNA collections as an assembly reference, and the high cost and tremendous throughput limits the number of samples that can be analyzed simultaneously, restricting the analysis to a small number of samples and only to model species.

Expressed Sequenced Tag (EST) sequencing using the Sanger technology has been extensively used to provide a first catalog of a species' gene inventory, and it is still the most used method to obtain transcriptome data for microarray construction. The main drawback of EST programs, in addition to being sensitive to cloning biases that affect which sequences are represented and how sequence-complete each clone is [14], is the tremendous effort in terms of cost and labor required to build up an extensive EST collection. As an alternative, next generation sequencing is being proposed as a technology for EST development in non- model organisms, even if sequences produced are just short reads which can difficultly be properly assembled in the absence of a genomic sequence to provide transcriptome characterization [15-17].

Here, we show that 454 sequencing of 3' cDNA fragments from a normalized library constructed from pooled RNAs provides a powerful method to rapidly build up an extensive catalog of unique transcripts that can be successfully used to develop a microarray for large scale analysis of gene expression in any species, without the need for prior sequence knowledge. As proof of principle, we evaluated the potential of this method for expression profiling during berry maturation in grape as if no other sequence information was available for this species. We then took advantage of the availability of the grape genome sequence and the extensive EST collection, to assess the quality of the catalog of transcripts produced. Finally, we evaluated the performances of a custom microarray derived from this catalog, and found that this microarray was more informative than one of the most comprehensive grape microarrays available to date, the GrapeArray 1.2 that we developed within the Italian-French Public Consortium for Grapevine Genome Characterization.

## Results

### Gene discovery using 454 sequencing

A pool of RNA extracted from grape berries harvested at different phenological stages was used to prepare a non-normalized (NN) and a normalized (N) cDNA library. In order to sequence the corresponding 3' mRNA ends, cDNAs from both libraries in the range of 450-550 bp were sequenced from the 5' end to retain the directional orientation of the reads and minimize the degrading performances of pyrosequencing technology when sequence extension reaches the poly A tail.

**Figure 1:**
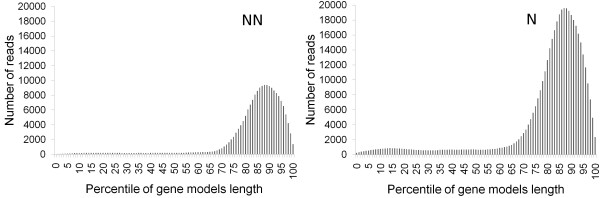
**Pyrosequencing reads represent the 3' end of *Vitis vinifera *transcripts**. The position of 9,366 reads from the non-normalized (NN) 454 library matching 1,948 gene models, and the position of 21,512 reads from the normalized (N) 454 library matching 3,749 gene models, expressed as a percentile of the length of the gene model to which the read mapped.

A total of 556,742 high quality reads was obtained from two halves of a 454 GS FLX run, one for each of the two libraries. Processing of the raw sequences to remove adaptors and, if present, the polyA, resulted in 290,167 reads from the NN library, and 266,575 reads from the N library, totaling 127 Mbp of grape expressed sequences. Mapping of the reads to grape gene models [18] of at least 1,000 nt in length, confirmed that the two libraries were representing the 3' ends of transcripts (Figure 1).

**Figure 2:**
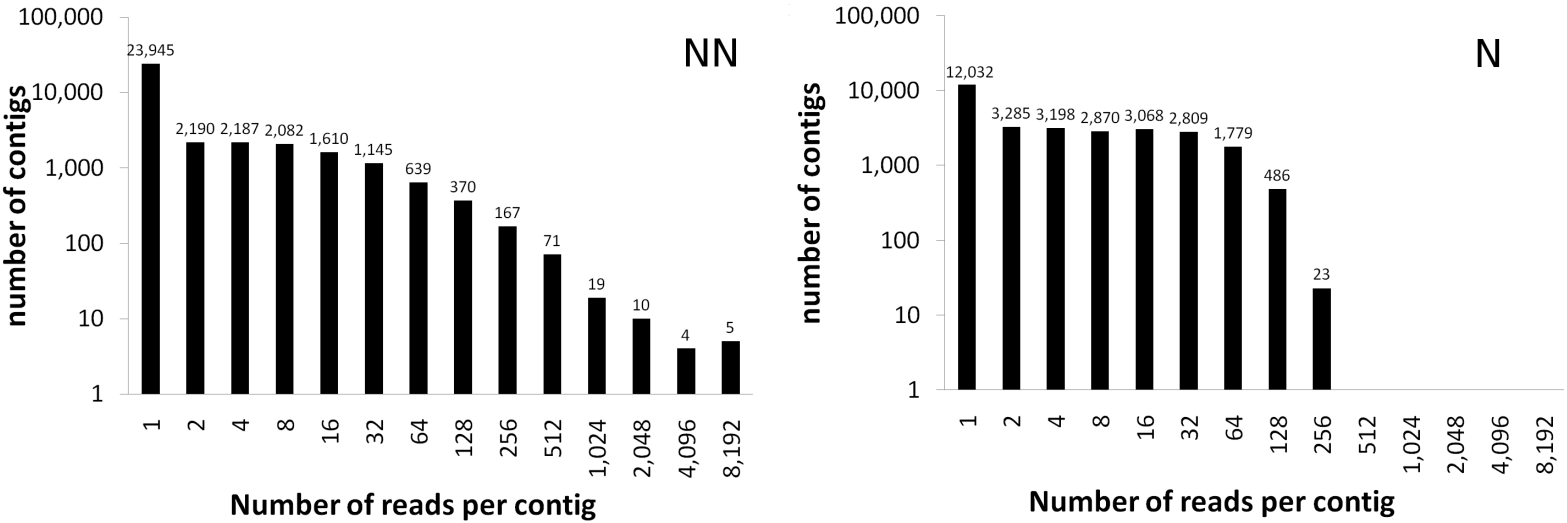
**Number of reads per contig in the non-normalized (NN) and normalized (N) libraries**. For each library, the number of contigs presenting the indicated amount of reads is plotted as a histogram and reported as a label for each histogram.

The two sets of reads entered the "de novo assembly" process using the Newbler software (Table 1). For the NN library, 90% of the reads were assembled in 10,532 contigs of 234 bp average length. The remaining 23,945 reads (coverage depth = 1) longer than 100 bp were retained as singletons, for a total of 34,477 unigenes. Normalization of the library increased the reads assembled to 95%, boosted the number of contigs to 17,595 and lowered their average depth of coverage from 24 to 15 reads per contig (Figure 2). The average contig length was 239 bp, therefore similar to that of the NN library. There were 12,032 singletons longer than 100 bp, totaling 29,627 unigenes.

**Table 1:** Summary statistics of *de novo *assembly.

Library	Total Contigs	Assembled Reads	Average contig length	Average depth	Singletons	Unigenes	Coverage (bp)
**NN**	10,532	261,702	234 bp ± 105 (s.d.)	24	23,945	34,477	8,501,963
**N**	17,595	252,935	239 bp ± 124 (s.d.)	15	12,032	29,627	8,066,328

Statistics of *de novo *assembly of 454 reads from the non-normalized (NN) and normalized (N) libraries. Unigenes number is defined as the sum of contigs plus singletons.

### Design of oligonucleotide probes on the 454-derived unigenes

The two unigene sets entered the process for oligonucleotide probe design using Oligoarray 2.1 software [19]. The Oligoarray software computes oligonucleotide specificity by searching for similar sequences in a database containing all the transcribed sequence derived from the genome. When the genome sequence is not known, the Blast database that is used to compute oligo's specificity is represented by the set of transcribed sequences often derived from the expressed sequence tags (EST) available. For proof of principle purposes, the BLAST database that we used was built only with the two set of 454-derived unigenes, as if no other information for this species was available.

The probe design on the 34,477 NN unigenes produced 17,843 specific oligonucleotide sequences (Table 2). The high number of NN unigenes for which a specific oligonucleotide probe could not be designed was mainly due to the presence in this library of highly redundant singletons not assembled by the software, as 13,688 NN singletons were mapping to just 10 genes models (data not shown). The number of unigenes in the NN library was therefore overestimated, but the Oligoarray software successfully recognized their redundancy. The probe design on the 29,627 N unigenes produced 29,393 specific oligonucleotide sequences (Table 2). By comparison, the probe design on the 33,638 TCs from the VvGI produced 32,771 oligonucleotides.

**Table 2:** Oligonucleotide probes design.

	NN	N	TCs
Unigenes	34,477	29,627	33,638

**Designed oligonucleotide probes**	**17,843**	**29,393**	**32,771**
Probes specific for 1 sequence	16,990	28,187	25,658
Probes recognizing 2-3 sequences	801	1,177	6,326
Probes recognizing 4-5 sequences	52	29	787

**Oligonucleotide probes mapping to genome**	**16,500**	**25,879**	**26,578**
Probes mapping to a unique position	14,146	21,869	21,831
Probes mapping to a 2-3 positions	1,594	2,654	2,848
Probes mapping to 4-5 positions	301	506	566
Probes mapping to more than 5 positions	459	850	1,333
Probes not mapping to genome	1,343	3,514	6,193

Oligonucleotide probes design was performed on the unigenes identified in the non-normalized (NN) and normalized (N) libraries and on the TCs comprised in VvGI.

### Mapping of 454-derived unigenes and of designed oligonucleotides to the genome sequence

To assess the use of 454-derived unigenes to develop an extensive catalog of unique transcripts without the need for prior sequence knowledge, the two sets of unigenes were mapped to the assembled grape genome sequence [18] using the BLAT algorithm, which takes into account introns by allowing the splitting of unigenes (Table 3). Using a stringent threshold of 95% coverage and 95% identity, 90% of the unigenes for the NN library, and 84% for the N library, matched to the grape genome. The BLASTX analysis of the unigenes that did not map to the genome revealed that 799 (NN) and 1,390 (N) of them had a match (threshold 1 × E^-7^) against the Uniprot plant database http://www.uniprot.org/downloads. Interestingly, 18 NN unigenes not mapping to the genome and without significant matches to plant sequences, had a significant match (1 × E^-10^) to *Botrytis cinerea *predicted genes or transcripts http://www.broad.mit.edu/annotation/genome/botrytis_cinerea. This number increased to 956 (mainly singletons) for the N unigenes, confirming that normalization significantly improved the proportion of low abundant sequences.

**Table 3:** Mapping of unigenes to the grape assembled genome sequence.

Unigenes	Unique position	Multiple positions	Not mapping
NN	29,838	1,201	3,438
N	22,900	1,925	4,802
TCs	22,436	1,749	9,453

Mapping statistics to genome of sequences from each dataset (non-normalized (NN) unigenes, normalized (N) unigenes and TCs from VvGI).

For comparison, the analysis of the whole set of 33,638 tentative consensus (TC) sequences assembled from 347,879 expressed sequence tags (EST) included in the release 6.0 of the *Vitis vinifera *Gene Index (VvGI), revealed that 72% of them could be mapped to the grape genome. Therefore, the 454-derived unigenes map a genome location better than assembled ESTs produced by conventional sequencing.

To evaluate the specificity of the oligonucleotide probes designed on the two sets of 454-derived unigenes, we then performed a Blast analysis of the probes against the grape genome (Table 2). The percentage of oligonucleotides mapping to a unique location was 79% for the NN library, and 74% for the N library. By comparison, the percentage of oligonucleotides designed on the TCs from the VvGI mapping to a unique location was 67%. Thus, the specificity of 454-derived oligonucleotides was higher than for the oligonucleotides designed on the extensive collection of ESTs available for this plant.

### Transcriptome representation of the 454-derived oligonucleotide probes

The grape gene models dataset contains 30,434 predicted gene loci [18]. To assess how the 454-derived oligonucleotide probes represent the grape transcriptome, we compared the genomic map position of the unigenes for which a specific oligonucleotide probe was designed, with the map positions of the gene loci. We could not directly compare oligonucleotide probes to grape predicted cDNAs as oligonucleotide probes were designed preferentially on the 3' untranslated region, which is not yet included in 45% of grape predicted cDNAs (data not shown).

The 17,843 NN unigenes for which a specific oligonucleotide probe was designed, matched 10,798 coding sequences. As limitations still affect the grape genome annotation, we extended the co-localization analysis to introns and to regions up to 500 bp downstream of the coding sequences [20], increasing the number of genes represented to 11,979. Mapping the other unigenes matching the grape genome in regions not annotated as coding sequences to the VvGI identified a further 2,159 grape transcripts. The remaining 1,468 unigenes mapping unannotated regions of the grape genome were considered putative novel genes [21]. In total, the set of NN unigenes represented 15,606 grape genes (Table 4).

**Table 4:** Alignment statistics of 454 unigene transcriptome catalogs and VvGI 6.0 TCs with an oligo

	NN	N	TC
Sequences with oligonucleotide probe designed	**17,843**	**29,393**	**32,771**
			
Gene loci matches			
- Within exons	10,798	14,176	15,552
- Within introns	808	939	775
- 500 bp downstream	373	359	82
			
VvGI 6.0 matched by sequences mapping to unannotated genome regions	2,159	2,663	2,989
Putative new genes (sequences mapping to unannotated genome only)	1,468	3,709	**-**
Total number of grape transcripts identified	**15,606**	**21,846**	**19,398**

454 unigene transcriptome catalogs with a designed oligo and VvGI 6.0 TCs with an oligo were aligned to known gene loci, unannotated genomic regions and ESTs. Number of gene loci, ESTs and putative novel genes, identified by all sequences mapping to grape genome in the three different libraries considered (non-normalized (NN), normalized (N), and TCs comprised in VvGI) are given.

For the N library, the 29,393 unigenes for which a specific oligonucleotide probe was designed corresponded to 14,176 grape gene loci. The inclusion of introns and the 500 bp region downstream of the coding sequences increased the grape genes identified to 15,474. Mapping the unigenes matching the grape genome in regions not annotated as coding sequences to the VvGI identified a further 2,663 transcripts that, together with the remaining 3,709 unigenes mapping to unannotated regions of the genome and that we considered putative novel genes, raised the grape genes represented to 21,846.

By comparison, the 32,771 TCs for which a specific oligonucleotide probe was designed, corresponded to 19,398 grape genes (Table 4). Therefore, the transcriptome representation of unigenes obtained from the N library is comparable to - if not higher than - that given by the TCs from the VvGI.

### Performance of microarray probes

A Combimatrix microarray carrying the two sets of oligonucleotide probes designed on the NN and N unigenes was then hybridized with the same pool of RNA used for the construction of the two 454 libraries (Additional file 1). For the NN set, 16,840 oligonucleotide probes corresponding to 14,115 genes produced hybridization signal intensities above the threshold, calculated as the mean plus two standard deviations of the negative reference samples [22], and confirmed the expression for 1,251 of the 1,468 putative novel genes (Additional file 2). For the N set, 26,733 oligonucleotide probes corresponding to 19,609 genes produced hybridization signal intensities above the threshold, and confirmed the expression for 3,098 of the 3,709 putative novel genes (Additional file 2). Finally, we performed a microarray hybridization to the GrapeArray 1.2, the Combimatrix-based grape chip developed by the Italian-French Public Consortium for Grapevine Genome Characterization, which comprises 24,562 probes designed on the release 5.0 of the VvGI integrated with genes predicted from the grape genome [23]. On this chip, 19,395 probes corresponding to 15,556 genes produced hybridization signal intensities above the threshold (Additional file 2). These results definitively validate the quality of a microarray chip based on 454-derived unigenes, as well as the 3' enriched library construction method.

## Discussion

Due to the large number of reads afforded, the 454 DNA sequencing technology is effective in revealing the expression of a large number of genes and has a great potential for discovering many rare or novel transcripts [21] also in non-model organisms where few previous ESTs sequences are available [16]. Combining the pyrosequencing of pooled samples derived from tissues or conditions to be analyzed in detail with the generation of a specific microarray based on developed sequence information, therefore, has the potential for allowing large scale expression analysis of the majority of genes expressed in those tissues or conditions also in non-model organisms [15].

To date, random pyrosequencing of cDNAs is still unable to accomplish *de novo *assembly for a solid gene reconstruction and transcriptome characterization, and data produced by this approach are so far largely used in sequenced genomes to refine annotated gene structures or to propose novel gene models [14,21,24]. It has been suggested that 3'cDNA 454-sequencing can enable resolution of a catalog of unique transcripts, eliminating overestimation associated with shotgun sequencing of multiple non-overlapping 454-ESTs per transcript [20,25]. We therefore produced pooled libraries enriched for 3'cDNA ends in order to limit the number of contigs for the same transcript and, consequently, the redundancy of the probe sets. The specificity of 3'-UTR-based sequence reads should also facilitate unambiguous gene assignment and, consequently, it has the potential for allowing the identification and analysis of nearly identical paralogous genes, as previously demonstrated [25]. With this approach, the capacity of 454-derived unigenes to map to a unique location on the grape genome was very high, similar to or even better than that of the TCs comprised in the VvGI. It was also definitely higher than for unigenes identified by random 454 sequencing of cDNA [24]. Furthermore, as 454 reads were derived from only one strand, the resulting sequences have known directional orientation.

A challenge for any EST project is obtaining sufficient coverage of less abundant transcripts [24]. As the aim of this study was to maximize the number of genes represented in the 454-derived EST catalog, we evaluated the potential advantage of cDNA library normalization. In previous works this was applied both to model and non-model organisms and was recently reported that normalization could have little influence on the efficiency of gene discovery when working with thousand of reads from a single tissue type [17]. However, so far normalization has not been performed on 3'cDNA libraries used in 454 sequencing, as the aim of these studies was always to assess also relative gene expression. We observed here that normalization increased the number of contigs assembled, and reduced the average number of reads per contig, clearly limiting over-representation of abundant transcripts. Furthermore, normalization dramatically improved the sampling of rare transcripts, as revealed by the higher number of contaminant fungal sequences found in the N library.

To demonstrate the high quality of the information that can be obtained by this approach and that the information can successfully be used to build up a microarray, we compared the effectiveness of the 454-derived unigene sets for oligonucleotide probe design with that of the TCs obtained from the 33,638 TCs assembled from the 347,879 Sanger-based ESTs included in the release 6.0 of the *Vitis vinifera *Gene Index. With just half of a 454 sequencing run of a 3'-cDNA normalized library, we could develop a microarray that can recognize 21,846 genes (15,606 for the non-normalized library). By comparison, the microarray designed on the extensive collection of ESTs from the last release of the VvGI can recognize only 19,398 genes. It should be noted that the 454-derived microarrays also carry a number of probes targeting previously unknown genes, which are not represented in the VvGI nor they have been predicted from the assembled grape genome, thus revealing a high coverage depth of the grape transcriptome.

In fact, the microarray designed on the unigenes from the normalized library proved to be more informative than one of the most comprehensive grape microarrays available to date, the GrapeArray 1.2 developed by the Italian-French Public Consortium for Grapevine Genome Characterization. This was demonstrated by comparing the performances of the GrapeArray 1.2 with those of the two microarrays designed on the set of NN and N unigenes, in detecting the expression of genes during grape berry maturation, a phenomenon that we are extensively studying by cDNA-AFLP [26], microarray and deep sequencing analyses (unpublished), and that we have adopted as reference to compare the different expression profiling methodologies currently available. Hybridization with a pool of RNAs from grape berries revealed that the GrapeArray 1.2, which carries 24,562 probes, could detect the expression of 15,556 genes. By comparison, the microarray carrying 17,843 probes designed on the NN unigenes, detected the expression of 14,115 genes, 1,251 of which were previously unknown. Strikingly, the microarray carrying 29,393 probes designed on the N unigenes, detected the expression of 19,609 genes, 3,098 of which are novel. These data confirm the effectiveness of cDNA normalization in increasing the number of genes that can be identified, and show the effectiveness of the proposed method in allowing genome-wide microarray analyses also in species for which very limited gene information, if any, is available. We anticipate that adaptation to the Titanium upgrade of the 454 platform, which extends the average length of the sequences to about 400 bp and increases the number of reads per run to 1.2 millions, will further strengthen the power of this approach.

## Conclusion

The costs, the amount of data produced and moreover the problems related to assembly of short reads make it unlikely that next generation sequencing will replace microarrays in the short term as the routine tool for expression profiling, especially for all those organisms for which the complete genome is not available. For those, but as shown here, also for sequenced organisms, 454-based 3'-cDNA sequencing of a normalized pool of cDNAs represents a solid, cost-effective and fast method to build up a comprehensive catalog of strand-specific ESTs, overcoming most of the limitations of Sanger-based ESTs. Furthermore we here demonstrate that combining this approach with the set up of a microarray make it feasible extensive analysis of gene expression and functional genomics studies, and that this approach can successfully be applied also to non model organisms.

## Methods

### Preparation of a non-normalized and a normalized cDNA library from berry skins of *V. Vinifera*

Berries of *V. vinifera *cv Corvina (clone 48) were harvested at 6 different time-points (Additional file 1) from veraison to withering over the course of the 2005 growing season from an experimental vineyard in the Verona Province (San Floriano, Verona, Italy). 30 clusters were collected for each sampling time-point, and 12 berries were sampled from each cluster to form a pool for each time-point. Total RNA was extracted according to [27] and equal quantities of total RNA were pooled together for a total of 300 ug of total RNA.

The cDNA libraries were prepared by Eurofins MWG Operon, Ebersberg, Germany http://www.eurofinsdna.com in cooperation with Vertis Biotechnologie AG, Freising, Germany. In short: after enrichment of polyA+ RNA, the first strand cDNA was synthesized using an oligo(dT)-adapter primer. After purification of the first strand cDNA, second strand cDNA synthesis was performed using a random (N)_6_-adapter primer. The non-normalised cDNA was amplified with 15 cycles of PCR using a high fidelity DNA polymerase.

With an aliquot of the cDNA, normalization was carried out by one cycle of denaturation and reassociation of the cDNA (cot curve). The reassociated double strand cDNA was separated from the remaining single strand cDNA (i.e. the normalized cDNA) by passing the mixture over a hydroxyl apatite column. After hydroxyl apatite chromatography, the single stranded cDNA was subjected to 8 PCR cycles.

For both libraries, fragments in the 450 - 550 bp size range were eluted from preparative agarose gels. An aliquot of the size fractionated cDNA was analyzed on a 1.5% agarose gel. Both cDNA libraries have a size of approx. 450 - 550 bp and the following structure:

*GCCTCCCTCGCGCCATCAG***+ACTACTGGAACCGACAGTGAGTA**+(NNNNNNNNNNNNNNNNNNNNNNNNNNNN)_(400~500 nt)_+AAAAAAAAAAA+**CTTCTCGTCCTCTGCCTGATTAGT**+*CTGAGCGGGCTGGCAAGGC*

454 Adapter A and B underlined, library specific 5'-Adapter and 3'-Adapter in bold.

Sequencing was performed on a Genome Sequencer GS FLX Instrument (Roche Diagnostics) following standard protocols [28].

### Bioinformatics and data analysis

454 reads were first quality filtered with standard parameters and Raw reads were cleaned from adaptor sequences. Enrichment of 3'-ends of transcripts was verified by mapping 454 reads against grape gene models (Additional file 3). Files containing 454 reads and their quality scores are available from the National Center for Biotechnology Information (NCBI) Short Read Archive [GenBank: accession number SRA007722]. Sequences were then assembled *de novo *into contigs using Newbler v1.1 [29] set with parameters shown in Additional file 4. Only assembled contigs longer than 100 bp were considered. Mapping of 454 sequences (contigs and singletons), VvGI 6.0 TCs and GrapeArray 1.2 target sequences to grape genome assembly 8.4X http://www.genoscope.cns.fr/externe/Download/Projets/Projet_ML/data/assembly/goldenpath/unmasked/ and to VvGI release 6,0 http://compbio.dfci.harvard.edu/cgi-bin/tgi/gimain.pl?gudb=grape was performed with BLAT [30]. Assignment of 454 unigenes, VvGI 6.0 TCs and GrapeArray 1.2 targets to grape annotated gene models was performed using custom scripts (Additional file 3). Translated BLAST [31] searches (BLASTX) of 454 unigenes against Uniprot release 14 database http://www.uniprot.org were performed with an e-value cutoff set at 1 × 10^-7^. 454 unigenes were compared to *Botrytis cinerea *transcripts and predicted genes http://www.broad.mit.edu/annotation/genome/botrytis_cinerea using an e-value cutoff set at 1 × 10^-10^. Oligos were aligned to genomic sequences with BLAST. Only alignments with at least 70% coverage and maximum 2 mismatches were considered. All sequences, blat and blast results, annotation tables were loaded with custom python scripts into a MySQL database. A graphical visualization of mappings is available at web address http://ddlab.sci.univr.it/cgi-bin/gbrowse/grape/; username "anonymous" and password "ye6Upraq"). Oligonucleotide probes were designed for 454 unigene sequences (contigs and singletons) from both libraries (N and NN) and for TC sequences (VvGI 6.0, http://compbio.dfci.jarvard.edi/tgi/cgi-bin/tgi/gimain.pl?gudb=grape) using OligoArray 2.1 software [19] (see Additional file 5). Details of oligo design and selection are described in Additional file 3.

### Microarray construction and hybridization

Custom 90K CombiMatrix arrays were prepared with the oligonucleotide sequences designed on the 454-derived unigenes using a CustomArray Synthesizer (CombiMatrix, Mulkiteo, USA). A more detailed description of the process for preparation of microarrays is shown in Additional file 3. Five μg of the same pool of total RNA used for 454 sequencing were labeled using RNA Ampulse amplification and labeling kit with Cy5 for Combimatrix arrays (Kreatech Diagnostics, The Netherlands) according to manufacturer instructions, and were hybridized to arrays according to CombiMatrix protocols. Scanning was performed on a GenePix 4000B scanner. Data extraction was done using CombiMatrix Microarray Imager software and a quantile normalization of data was performed using Combimatrix Blist v0.6 software. A gene was considered expressed when the corresponding probe had signal intensity above the threshold, calculated as the mean plus two standard deviations of the negative reference samples [22]. Expression data are available from the National Center for Biotechnology Information (NCBI) [GenBank: Gene Expression Omnibus accession number GSE14276].

## Authors' contributions

DB, AF and AC conducted the experimental procedures and sequence analysis. OK performed the 454 pyrosequencing and NL did the reads assembling. PB designed and organized the 454 proof of principle. MD participated in the 454 proof of principle design and wrote the manuscript. All authors contributed to the content of the manuscript and have read and approved the final version.

## Supplementary Material

Additional file 1 **Collection date and development stage of berry samples analyzed**. Collection date and development stage of berry samples used to constitute the RNA pool analyzed. Click here for file

Additional file 2 **Alignment statistics of positive 454 unigene sequences and positive sequences used to design the GrapeArray 1.2**. Alignment statistics of 454 unigene sequences with a positive expression call by microarray analysis and sequences used to design the GrapeArray 1.2 with positive call by microarray to known gene loci, unannotated genomic regions and ESTs. Number of gene loci identified, ESTs identified and putative novel genes identified by all sequences mapping to grape genome in the three different libraries considered are given. Click here for file

Additional file 3 **Additional Methods**. Additional information for the Methods section. Click here for file

Additional file 4 Parameters used for the *de novo *assembly of single reads with Newbler v1.1 software. Click here for file

Additional file 5 Parameters used for oligo design with OligoArray 2.1. Click here for file
