# Supplementary material for: Combining next-generation pyrosequencing with microarray for large scale expression analysis in non-model species
Source: BMC Genomics. 2009 Nov 24;10:555. doi: 10.1186/1471-2164-10-555 (PMC2790472; doi:10.1186/1471-2164-10-555)
Supplement: Additional file 1 — Collection date and development stage of berry samples analyzed. Collection date and development stage of berry samples used to constitute the RNA pool analyzed. [file 1471-2164-10-555-S1.DOC]

| **Sample** | **Collection date** |
| --- | --- |
| Véraison | 03/08/05 |
| Ripening I | 22/08/05 |
| Ripening II | 15/09/05 |
| Withering I | 18/10/05 |
| Withering II | 17/11/05 |
| Withering III | 14/12/05 |
