# Supplementary material for: Combining next-generation pyrosequencing with microarray for large scale expression analysis in non-model species
Source: BMC Genomics. 2009 Nov 24;10:555. doi: 10.1186/1471-2164-10-555 (PMC2790472; doi:10.1186/1471-2164-10-555)
Supplement: Additional file 2 — Alignment statistics of positive 454 unigene sequences and positive sequences used to design the GrapeArray 1.2. Alignment statistics of 454 unigene sequences with a positive expression call by microarray analysis and sequences used to design the GrapeArray 1.2 with positive call by microarray to known gene loci, unannotated genomic regions and ESTs. Number of gene loci identified, ESTs identified and putative novel genes identified by all sequences mapping to grape genome in the three different libraries considered are given. [file 1471-2164-10-555-S2.DOC]

|  | **NN** | **N** | **GrapeArray 1.2** |
| --- | --- | --- | --- |
| Oligos with a signal > background + 2 standard deviations | 16,840 | 26,733 | 19,395 |
| Gene loci matches: |  |  |  |
| - Within exons | 10,531 | 13,649 | 13,100 |
| - Within introns | 748 | 833 | 597 |
| - 500 bp downstream | 366 | 336 | 81 |
| VvGI 6.0 matched by sequences mapping to unannotated genome regions | 1,219 | 1,713 | 1,778 |
| Putative new genes (sequences mapping to unannotated genome only) | 1,251 | 3,078 | -- |
| Total number of grape transcripts identified | 14,115 | 19,609 | 15,556 |
