# Supplementary material for: Combining next-generation pyrosequencing with microarray for large scale expression analysis in non-model species
Source: BMC Genomics. 2009 Nov 24;10:555. doi: 10.1186/1471-2164-10-555 (PMC2790472; doi:10.1186/1471-2164-10-555)
Supplement: Additional file 3 — Additional Methods. Additional information for the Methods section. [file 1471-2164-10-555-S3.DOC]

# Additional Methods

## Distribution of 454 reads across length of gene models

Distribution of 454 reads across length of gene models was evaluated by mapping 454 reads against *Vitis vinifera* gene models (http://www.genoscope.cns.fr/externe/Download/Projets/Projet_ML/data/annotation/Vitis_vinifera_mRNA_v1.fa). As an example 1,948 gene models of 1,000-2,000 bp with 1 to 201 reads were selected for NN library and 3,749 gene models of 1,000-2,000 bp with 1 to 101 reads per gene model were selected for N library. Mapping was performed by BLAT alignment and resulting data tables were filtered with a custom python script using stringent parameters of 95% sequences identity over at least 95% of sequence length. Gene models were subdivided into 100 percentiles and position of each read alignment was expressed as percentile of the length of the gene model to which it mapped.

## Mapping of 454 unigenes to grape annotated gene models

In order to assign identities between 454 unigenes, VvGI TCs and predicted gene models, sequences mapping coordinates to genome (http://www.genoscope.cns.fr/externe/Download/Projets/Projet_ML/data/assembly/goldenpath/unmasked/), obtained with BLAT, were compared to gene models mappings on grape 8.4x assembly. Before comparison BLAT results were filtered with a custom python script using stringent parameters of 95% sequence identity over at least 95% of sequence length. Gene models annotations were downloaded from Genoscope public repository (http://www.genoscope.cns.fr/externe/Download/Projets/Projet_ML/data/annotation/Vitis_vinifera_annotation_v1.gff) and compared to 454 unigenes mappings, by using custom python scripts. 454 unigenes mappings were first assigned to exons; for gene models for which no 454 unigene mapping to exons was detected, mappings to introns were analyzed and finally 454 sequences mapping to regions up to 500 bp downstream gene models predictions were assigned. 454 unigenes not mapping to cds exons, introns or to regions 500bp downstream gene models but having a match on genome were mapped to VvGI 6.0 transcribed sequences by BLAT alignment. The same strategy used for 454 unigenes was used also to assign VvGI TCs.

## Microarray design and preparation

Parameters for oligo design (Additional file 5) were set in order to be compatible with CombiMatrix protocols (http://www.combimatrix.com/support_docs.htm). Oligo designed on 454 unigene sequences and TCs were checked for cross-hybridization by BLAST searches against all 454 unigene sequences and TCs respectively. Oligos with more than 5 cross-hybridizations were removed with a custom python script to avoid biases due to redundancies during normalization procedures. Oligos designed on 454 unigene sequences of NN and N library were assembled in a unique dataset and redundancies between the two datasets were removed (44,156 probes left). Chip layout was designed using Layout Designer 4.2.1 CombiMatrix software (CombiMatrix, Mulkiteo, USA). A total number of 2 replicates was set for each probe and replicates were randomly distributed on the chip. In addition to standard CombiMatrix negative and quality controls included by default in factory layouts, 10 negative controls designed on bacterial and viral sequences (*Bacillus anthracis* phage Gamma genome, *Haemophilus ducreyi* genome, *Alteromonas* phage PM2 genome) were added in 10 replicates randomly distributed on the chip. Negative control probes were designed using the same parameters used for design of oligos on 454 unigenes (Additional file 5) and were checked for cross-hybridization by BLAST searches against all 454 sequences. Microarrays were prepared on Blank 90K CustomArray chips using a 90K/12K CombiMatrix Synthesizer (CombiMatrix, Mulkiteo, USA) with the standard manufacturing and quality control protocols provided from CombiMatrix.
