# Supplementary material for: Combining next-generation pyrosequencing with microarray for large scale expression analysis in non-model species
Source: BMC Genomics. 2009 Nov 24;10:555. doi: 10.1186/1471-2164-10-555 (PMC2790472; doi:10.1186/1471-2164-10-555)
Supplement: Additional file 4 — Parameters used for the de novo assembly of single reads with Newbler v1.1 software. [file 1471-2164-10-555-S4.DOC]

| **Parameter** | **Value** |
| --- | --- |
| Minimum reads overlap length | 40bp |
| Minimum identity of overlapping reads | 90% |
| Seed step | 12bp |
| Seed length | 16bp |
