# Supplementary material for: Combining next-generation pyrosequencing with microarray for large scale expression analysis in non-model species
Source: BMC Genomics. 2009 Nov 24;10:555. doi: 10.1186/1471-2164-10-555 (PMC2790472; doi:10.1186/1471-2164-10-555)
Supplement: Additional file 5 — Parameters used for oligo design with OligoArray 2.1. [file 1471-2164-10-555-S5.DOC]

| **Parameter** | **Value** |
| --- | --- |
| Oligonucleotide length range | 35-40bp |
| Melting temperature (Tm) range | 80-86 °C |
| Secondary structures temperature threshold | 70 °C |
| Cross-hybridization threshold temperature | 65 °C |
| GC content range | 35-60% |
